# Hippocampal interictal discharges induce frontal spindles and enhance spindle–slow oscillation coupling in temporal lobe epilepsy

**DOI:** 10.1016/j.cnp.2026.04.005

**Published:** 2026-04-08

**Authors:** Taira Uehara, Ela Austria Barcelon, Hiroshi Shigeto, Takahiko Mukaino, Toshiki Okadome, Nobutaka Mukae, Ayumi Sakata, Hiroyuki Murai, Shozo Tobimatsu, Noriko Isobe, Jun-ichi Kira

**Affiliations:** a Department of Neurology, Neurological Institute, Graduate School of Medical Sciences, Kyushu University, Fukuoka, Japan; b Department of Clinical Neurophysiology, Neurological Institute, Graduate School of Medical Sciences, Kyushu University, Fukuoka, Japan; c Department of Neurology, International University of Health and Welfare, Narita, Japan; d Division of Medical Technology, Department of Health Sciences, Graduate School of Medical Sciences, Kyushu University, Fukuoka, Japan; e Department of Neurosurgery, Neurological Institute, Graduate School of Medical Sciences, Kyushu University, Fukuoka, Japan; f Department of Neurosurgery, Aso Iizuka Hospital, Iizuka, Japan; g Department of Clinical Laboratory, Kyushu University Hospital, Fukuoka, Japan; h Department of Orthoptics, Faculty of Medical Science, Fukuoka International University of Health and Welfare, Fukuoka, Japan; i Translational Neuroscience Research Center, Graduate School of Medicine, International University of Health and Welfare, Okawa, Japan; j Department of Neurology, Brain and Nerve Center, Fukuoka Central Hospital, International University of Health and Welfare, Fukuoka, Japan

**Keywords:** Focal epilepsy, memory impairment, accelerated long-term forgetting, sleep spindle, memory consolidation

## Abstract

**Objective:**

To investigate how hippocampal interictal epileptiform discharges (IEDs) influence cortical spindles, slow oscillations (SOs), and spindle–SO coupling, which are key components of sleep-dependent memory consolidation.

**Methods:**

Simultaneous intracranial and scalp EEG was recorded during NREM sleep in 10 patients with temporal lobe epilepsy (TLE). Hippocampal IEDs were identified using depth electrodes, while spindles and SOs were detected from ipsilateral scalp EEG in frontal, central, parietal, and temporal regions. Peri-IED time histograms and spectral analyses were performed to evaluate the temporal associations between IEDs and the oscillatory events. Spindle–SO coupling was assessed using phase consistency and amplitude modulation metrics.

**Results:**

Spindle occurrence increased in the frontal region 0.4–0.8 s after IEDs. This increase occurred regardless of their coincidence with SOs. The incidence of SOs increased within ± 0.4 s of IEDs across regions. Spectral analysis confirmed elevated frontal spindle-band power following IEDs. Phase consistency and amplitude modulation of spindle–SO coupling were higher for IED-coupled than for uncoupled spindles in the frontal region.

**Conclusions:**

Hippocampal IEDs selectively induced frontal spindles and enhanced their coupling with SOs.

**Significance:**

These findings suggest that IEDs may alter physiological hippocampal–cortical communication relevant to memory consolidation in TLE.

## Highlights

• Hippocampal interictal epileptiform discharges (IEDs) in temporal lobe epilepsy selectively increased frontal spindles.
• The spindle increase occurred regardless of their temporal coincidence with slow oscillations (SOs).
• IED-coupled spindles exhibited stronger coupling with SOs than did uncoupled spindles.

## 1 Introduction

Memory impairment is a major complication that can be even more debilitating than seizures, significantly affecting the quality of life in patients with epilepsy (de la Loge et al., 2016). Accelerated long-term forgetting (ALF), characterized by the rapid loss of episodic memories over days to weeks, is a well-documented form of memory disturbance, particularly in individuals with temporal lobe epilepsy (TLE) (Blake et al., 2000, Butler and Zeman, 2008). Previous studies have investigated various factors associated with ALF, including seizures, interictal epileptiform discharges (IEDs) (Butler and Zeman, 2008, Lambert et al., 2020), hippocampal atrophy (Mukaino et al., 2022), medication use, and psychological disorders.

Among these, hippocampal IEDs have been closely linked to ALF; however, the mechanisms underlying this association remain elusive (Lambert et al., 2021). ALF is considered a disorder of long-term memory consolidation. This process includes systems consolidation, in which memory traces are gradually transferred from the hippocampus to distributed neocortical networks. During non-rapid eye movement (NREM) sleep, the coordination of three key neural oscillations—hippocampal ripples, thalamocortical spindles, and neocortical slow oscillations (SOs)—is believed to play a critical role in systems consolidation (Geva-Sagiv et al., 2023, Helfrich et al., 2021, Klinzing et al., 2019, Rasch and Born, 2013).

Among these oscillations, spindles show temporal correlations with IEDs (Ferrillo et al., 2000, Nobili et al., 2000, Nobili et al., 1999, Zubler et al., 2017), but their precise relationship remained unknown until recently. In a seminal animal study, Gelinas et al. (2016) demonstrated a time-locked increase in prefrontal spindles following hippocampal IEDs in a TLE kindling model (Gelinas et al., 2016). These IED-induced spindles were associated with impaired overnight memory retention, suggesting that hippocampal IEDs may disrupt physiological hippocampal–cortical interactions critical for memory consolidation.

Despite this, whether hippocampal IEDs induce cortical spindles in human patients with TLE remains a matter of ongoing debate. While some reports have observed such effects in small cohorts (Dahal et al., 2019, Gelinas et al., 2016), a recent study concluded that the evidence remains inconclusive (Sákovics et al., 2022). Furthermore, some researchers have proposed that the observed IED–spindle coupling may simply reflect a shared modulation by SOs rather than a causal relationship (von Ellenrieder et al., 2020, Wodeyar et al., 2024), given that SOs are known to increase the incidence of both IEDs and spindles (Chen et al., 2023, Clemens et al., 2007, Frauscher et al., 2015b, Ujma et al., 2017, Wodeyar et al., 2024).

Even if hippocampal IEDs do induce spindles, key characteristics of these spindles, such as their spatial distribution and their coupling with SOs, remain poorly understood. A major barrier to progress in this area has been the lack of suitable datasets. Intracranial electroencephalography (EEG) is essential for accurately detecting hippocampal IEDs, while scalp EEG is necessary for assessing spindles and SOs across widespread cortical areas. To address this limitation, in this study, we employed simultaneous intracranial and scalp EEG recordings to investigate the relationships between hippocampal IEDs, cortical spindles, SOs, and spindle–SO coupling in patients with TLE.

## 2 Methods

### 2.1 Patients

We retrospectively reviewed the data from 11 consecutive patients with medically intractable TLE who underwent simultaneous depth and scalp EEG recordings during presurgical evaluations between December 2005 and October 2013 at Kyushu University Hospital. Intracranial recording was performed to aid lateralization in patients with presumed mesial TLE based on noninvasive evaluation (seizure semiology, ictal and interictal scalp EEG, and neuroimaging), when laterality remained uncertain (e.g., suspected bilateral onset or discordance between imaging lateralization and ictal scalp EEG lateralization). We excluded patients if seizures were too frequent to extract the predefined 60-min-long NREM sleep EEG data (see Data selection). We then selected the hemispheres for analysis using the following criteria: (1) hippocampal IED density was greater than 5/min, and (2) lack of continuous artifacts in the frontal (F3/4), central (C3/4), parietal (P3/4), and temporal (F7/8) electrodes. The Kyushu University Institutional Review Board for Clinical Research approved this study (permission number: 30–140). The requirement for informed consent was waived.

### 2.2 Acquisition of EEG data

**Fig. 1:**
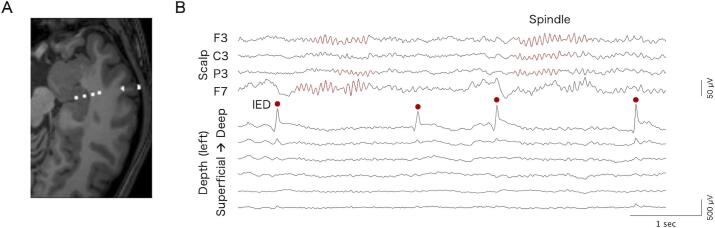
**Example of simultaneous intracranial and scalp electroencephalographic (EEG) recordings.** (A) Post-implantation computed tomography showing a depth electrode overlaid on pre-implantation magnetic resonance imaging in an example patient. (B) Representative EEG traces from the hippocampal depth electrode and ipsilateral scalp electrodes. Red circles indicate manually identified hippocampal interictal epileptiform discharges (IEDs). Automatically detected spindles in the scalp EEG are highlighted in red. (For interpretation of the references to colour in this figure legend, the reader is referred to the web version of this article.)

EEG data were obtained using the Nihon Kohden EEG system (Nihon Kohden, Tokyo, Japan) at a sampling rate of 500 (three patients) or 1000 (eight patients) Hz. High-frequency activity (e.g., high-frequency oscillations) was outside the scope of this study and was not analyzed; therefore, recordings acquired at 500 Hz were included in our analyses focusing on IED-related phenomena below 30 Hz. The reference was the average value recorded from the two scalp electrodes (C3 and C4). Bilateral hippocampal EEG recordings were acquired using two depth electrodes, one on each side, with six contacts implanted through the temporal cortex (Fig. 1A). The deepest contacts targeted the anterior hippocampus around the head–body junction. Mesial temporal lobe sampling with the depth electrodes was primarily limited to hippocampal contacts, and adjacent structures, including the amygdala, were not systematically sampled. Scalp electrodes were placed according to the international 10–20 system.

### 2.3 Data selection

For each patient, an expert electroencephalographer (AS), who was not involved in data analysis, selected six 5-minute artifact-free epochs from NREM sleep stage N2 and six 5-minute epochs from stage N3, totaling 60 min of data (30 min per stage). Sleep stages were determined every 30 s according to the criteria by the American Academy of Sleep Medicine. To minimize potential residual effects of anesthesia and early postoperative changes in sleep architecture, recordings from the first night after electrode implantation were not used for epoch selection. All stages during 1 h before and after any types of seizure were excluded from analysis. Two reviewers (EB and TU) meticulously inspected the scalp EEG recordings for artifacts. Electrodes exhibiting continuous artifacts were eliminated, and segments with transient artifacts in the remaining electrodes were excluded from analysis.

### 2.4 Detecting IEDs

The first reviewer (EB), a trained neurologist and electroencephalographer, manually identified the IEDs from the depth EEG recordings and marked their peaks. Subsequently, the second reviewer (TU), a board-certified neurologist and epileptologist, confirmed the results. IED identification was verified, if both reviewers agreed. IEDs were manually annotated on intracranial EEG using standard spike/sharp-wave morphology criteria, as summarized in the glossary of the International Federation of Clinical Neurophysiology (Kane et al., 2017). Specifically, IEDs were identified as transient events clearly distinguishable from background activity, with a pointed peak, a duration of 20–200 ms, and with or without a following slow wave. To standardize annotation, only events with an amplitude greater than three times that of background activity were considered IEDs. In this study, “hippocampal IEDs” were operationally defined as IEDs with the largest peak detected on the deepest two hippocampal contacts (Fig. 1B), which were used for subsequent analyses. Because adjacent mesial temporal lobe structures, including the amygdala, were not systematically sampled, this definition reflects IEDs recorded in hippocampal contacts rather than definitive source localization. We considered a sequence of multiple events fulfilling these criteria as a single IED, provided that there were no intervening background activities, and marked the highest peak. To avoid the inclusion of possible ictal discharges, those that lasted more than 1 s were excluded. Both reviewers were blinded to scalp EEG findings during the IED marking process.

### 2.5 Scalp EEG analysis

We analyzed the scalp EEG ipsilateral to the hippocampal IEDs in the frontal (F3/4), central (C3/4), parietal (P3/4), and temporal (F7/8) electrodes using MATLAB 2021b (MathWorks, Natick, MA, USA) with EEGLAB (https://sccn.ucsd.edu/eeglab/index.php) (Delorme and Makeig, 2004), and FieldTrip (https://www.fieldtriptoolbox.org) (Oostenveld et al., 2011) toolboxes.

#### 2.5.1 Detecting spindles and SOs

We automatically detected spindles and SOs from scalp EEG recordings using a previously employed method (Hahn et al., 2020, Helfrich et al., 2019, 2018). The mastoid is commonly used as a reference for the extraction of spindles and SOs. However, in our patient group, TLE, IEDs and pathological slow waves spilled over into the mastoid electrodes, thus making them inappropriate references. Therefore, in this study, we rereferenced the data to the average of the midline and parasagittal electrodes (Fp1, Fp2, F3, F4, C3, C4, P3, P4, O1, O2, Fz, Cz, and Pz) according to a previous study (Frauscher et al., 2015a). For spindle detection, the EEG signals were downsampled to 100 Hz and filtered between 12–16 Hz, and the envelope was extracted using a Hilbert transform and smoothed using a 200-ms moving average window. We then used continuous periods ranging between 0.5 and 3 s in which the amplitude of all data exceeded the 75th percentile of those in the artifact-free periods. For SO detection, the EEG recordings were downsampled to 100 Hz, high-pass filtered at 0.5 Hz, and low-pass filtered at 2 Hz, and positive to negative zero crossing time points were extracted. Subsequently, we designated an epoch between two consecutive time points as an SO, provided that its duration was between 0.8 and 2 s (corresponding to a range of 0.5 to 1.25 Hz) and its peak-to-peak amplitude exceeded the 75th percentile.

#### 2.5.2 IED–spindle and IED–SO coupling analysis

To investigate the relationship between hippocampal IEDs and scalp spindles or SOs, we constructed *peri*-IED time histograms for each hemisphere in each patient. These histograms counted the occurrence of spindle onsets or SO negative peaks within ± 4 s of a hippocampal IED peak, using 0.4-s time bins. Group-level statistical testing was performed to determine whether there was a consistent time lag between IEDs and spindles or SOs across patients.

To evaluate whether the observed IED–spindle coupling could be explained by the confounding influence of SOs, we further analyzed the data by separating IEDs into SO-unrelated and SO-related events. SO-unrelated IEDs were defined as those occurring at least 2.5 s away from any SO negative peak, whereas SO-related IEDs occurred within ± 2.5 s of an SO negative peak.

#### 2.5.3 Peri-IED spectral and amplitude analysis

As automatic spindle detection depends on the method chosen (Warby et al., 2014), we performed a *peri*-IED spectral analysis to supplement the IED–spindle coupling analysis. Using the Morlet wavelet transformation (seven cycles) implemented in FieldTrip, we calculated the power values of scalp EEG signals ± 2 s around hippocampal IED peaks, with 10-ms steps, across the frequency range of 1–30 Hz using increments of 1 Hz. To account for inter-individual EEG amplitude differences, power values were normalized to z scores using the mean and standard deviation across all analyzed recordings. Baseline activity, defined as the mean value between − 4 and − 2 s relative to IED peaks, was subtracted from each trial. Group-level statistical testing was then performed against zero. To complement the IED–SO coupling analysis, we similarly assessed *peri*-IED amplitude changes in the SO band. SO amplitude was extracted using a 0.5–1.25 Hz bandpass filter followed by Hilbert transformation applied to the ± 2 s window around IED peaks. The amplitude was z-score normalized, baseline-corrected using the − 4 to − 2 s interval, and tested against zero at the group level.

#### 2.5.4 Impact of IED on spindle and spindle–SO coupling

We evaluated whether hippocampal IEDs affect the features of scalp spindles. Spindles with a preceding hippocampal IED within 1 s were classified as IED-coupled and compared with other IED-uncoupled spindles with respect to frequency, amplitude, and duration. To account for inter-individual EEG amplitude differences, amplitude values were normalized to z scores. We also explored the relationship between IED density (counts per minute) and spindle density.

We further examined whether preceding IEDs affect the coupling between spindles and SOs. As conventional metrics such as phase-locking value (PLV) (Hahn et al., 2020, Helfrich et al., 2019, 2018) and modulation index (MI) (Tort et al., 2010) are sensitive to data length and not suitable for imbalanced data, we employed two alternative methods. The first was the spindles within preferred phase ratio (Hahn et al., 2020), a PLV-like metric that reflects the consistency of SO phases at which spindle peaks occur. SO phase angles were derived by applying a 0.5–1.25 Hz bandpass filter and Hilbert transform, and the preferred phase of spindle peaks in each hemisphere was identified using the CircStat toolbox in MATLAB. We then calculated the proportion of spindle peaks falling within ± 22.5° of the preferred phase and compared this ratio between IED-coupled and uncoupled spindles.

The second was an amplitude-based approach analogous to the MI method. Spindle amplitude was extracted using a 12–16 Hz bandpass filter and Hilbert transform from 2-second windows centered on spindle peaks. Amplitude values were z-score normalized within each hemisphere. Each window was divided into nine SO phase bins, and the average amplitude in each bin was calculated. We then assessed differences in SO phase–dependent amplitude modulation between IED-coupled and uncoupled spindles. Both analyses were limited to spindles occurring within 2.5 s of an SO negative peak.

We additionally evaluated the directionality of spindle–SO interactions using cross-frequency directionality (CFD) analysis based on the phase-slope index (PSI) (Helfrich et al., 2018, Jiang et al., 2015, Nolte et al., 2008). Spindle amplitude was obtained by bandpass filtering at 12–16 Hz followed by the Hilbert transform, and PSI was computed between this envelope signal and the original EEG signal. Fast Fourier transforms (FFTs) were computed in non-overlapping 4-s segments centered on the spindle peak, and PSI was derived from the frequency dependence of the phase difference within 0.5–1.25 Hz (frequency resolution of 0.25 Hz). Analyses were restricted to spindles occurring within ± 2.5 s of the SO negative peak and were performed separately for IED-coupled and IED-uncoupled spindles. PSI was normalized by the jackknife-estimated standard error to reduce the influence of differences in event counts. PSI is a directionality measure, and its sign indicates which signal precedes the other. In this analysis, the sign convention was set such that PSI was positive when changes in the SO-band EEG phase preceded fluctuations in spindle amplitude. PSI was computed using the MATLAB code provided in the original paper (Nolte et al., 2008).

#### 2.5.5 Impact of SOZ location on spindle and SO density

To explore whether seizure onset zone (SOZ) location influenced scalp spindle and SO density, each hemisphere was classified as mesial temporal SOZ, lateral temporal SOZ, or non-SOZ based on intracranial recordings. Spindle density and SO density were evaluated in the four ipsilateral scalp regions (frontal, central, parietal, and temporal). The primary exploratory comparison was between hemispheres with mesial versus lateral temporal SOZ.

### 2.6 Peri-IED hippocampal EEG analysis

IED-associated increases in cortical spindles may be accompanied by a concurrent increase in hippocampal spindles. To assess this, we analyzed hippocampal EEG time-locked to hippocampal IEDs. Hippocampal EEG was derived from a bipolar montage between the deepest two contacts of the depth electrode, and the same preprocessing as for scalp EEG (band-pass/notch filtering and resampling) was applied. In a preliminary analysis, we applied the same automated spindle detection used for scalp EEG; however, frequent IED-related false positives made the results unsuitable for quantification. Therefore, we did not perform spindle detection for hippocampal EEG. Instead, we limited the analysis to Morlet wavelet–based *peri*-IED spectral analysis to assess IED-related increases in spindle-band power. The *peri*-IED spectral analysis used the same settings and procedures as the scalp EEG analysis (Section 2.5.3).

### 2.7 Correlations between neuropsychological outcomes and IED–spindle or IED–SO coupling

To explore the relationship between IED–spindle/IED–SO coupling and cognitive function, we performed an exploratory analysis. Using the *peri*-IED time histograms generated as described in Section 2.5.2, each histogram was converted to z-scores based on its mean and standard deviation, and the maximum z-score within the time window of interest was used as an index of IED–spindle or IED–SO coupling strength. Based on the results of the primary analysis, the time window of interest was defined as 0 to 1.2 s after the IED for IED–spindle coupling and from 0.8 s before to 0.8 s after the IED for IED–SO coupling. When both hemispheres from the same patient were included in the analysis, the larger value was used as the representative patient-level index. As neuropsychological outcomes, we used preoperative full-scale intelligence quotient (FIQ), verbal intelligence quotient (VIQ), and performance intelligence quotient (PIQ) scores obtained from the Wechsler Adult Intelligence Scale-Revised (WAIS-R), Wechsler Adult Intelligence Scale-Third Edition (WAIS-III), or Wechsler Intelligence Scale for Children-Third Edition (WISC-III), and examined their associations with IED–spindle/IED–SO coupling strength. Memory-specific measures were not included because the neuropsychological test batteries differed across patients, precluding a unified analysis.

### 2.8 Statistical analyses

Statistical analyses were conducted using generalized linear mixed-effects models (GLMMs) and linear mixed-effects models (LMMs), as appropriate for the data, implemented with the lme4 package in R version 4.4.2 (R Foundation for Statistical Computing, Vienna, Austria). Degrees of freedom for LMMs were estimated using the Satterthwaite method. In all analyses, a *p* value < 0.05 was considered statistically significant.

For the *peri*-IED time histogram analysis, random intercept GLMMs were used to account for the repeated-measures design. Histograms were modeled using a Poisson distribution with a log link function. Fixed effects included the intercept and time bins, while random effects included hemisphere identity (“Hemisphere”) nested within patient identity (“Patient”). The overall effect of time bins was tested using a Type III Wald χ^2^ test. Multiple comparisons across electrode regions were corrected using the Bonferroni method. When the effect of time bins was statistically significant, post hoc comparisons were performed by testing estimated counts for each time bin against the mean across all bins using Wald tests in the emmeans package of R, with Bonferroni correction applied across time bins.

For the *peri*-IED spectral and amplitude analysis, baseline-subtracted power or amplitude values were analyzed using LMMs without averaging within each hemisphere, due to variable numbers of IEDs across hemispheres (Yu et al., 2022). Fixed effects included the intercept, and random effects included “Hemisphere” nested within “Patient”. Coefficients were tested against zero using *t*-tests. Multiple comparisons were corrected using the false discovery rate (FDR) method (Benjamini and Hochberg, 1995), applied across time points and regions, as well as across frequencies for spectral analyses.

Spindle features (frequency, amplitude, and duration) were also analyzed using random intercept LMMs without hemisphere-level averaging. Fixed effects included the intercept and spindle type (IED-coupled vs. uncoupled), while random effects included “Hemisphere” nested within “Patient”. For spindle density analysis, random intercept LMMs were used with fixed effects for the intercept and log-transformed IED density, and “Patient” as a random effect. Bonferroni correction was applied for comparisons across electrode regions.

To compare the spindles within preferred phase ratio between IED-coupled and uncoupled spindles, we used a GLMM with a binomial distribution and a logit link function. Fixed effects included the intercept and spindle type, and random intercepts were included for “Hemisphere” nested within “Patient”. To assess whether the spindle amplitude modulation by SO phase differed between IED-coupled and uncoupled spindles, we used a random intercept LMM. The dependent variable was z-scored spindle amplitude. Fixed effects included the intercept, SO phase bins, spindle type, and their interaction (SO phase × spindle type). Random intercepts were specified for spindle identities nested within “Hemisphere”, which was nested within “Patient”. A Type III ANOVA was performed to assess the interaction effect, with Bonferroni correction applied across electrode regions. If the interaction was statistically significant, pairwise comparisons between spindle types were conducted within each SO phase bin, with Bonferroni correction applied across bins. For the CFD analysis, PSI values were tested against zero using random-intercept LMMs with an intercept-only fixed effect and “Patient” as a random effect. Bonferroni correction was applied for multiple comparisons across regions.

To assess the effect of SOZ location, spindle density and SO density were modeled separately using random intercept LMMs with SOZ category (mesial temporal or lateral temporal) as a fixed effect and “Patient” as a random effect. Bonferroni correction was applied for comparisons across scalp regions.

For the exploratory analysis of neuropsychological correlates, associations between patient-level IED–spindle/IED–SO coupling strength indices and neuropsychological measures were assessed using Pearson’s correlation coefficients. Multiple comparisons across regions were corrected using the Bonferroni method.

## 3 Results

### 3.1 Patient characteristics

Ten patients were included in the study, whereas one was excluded owing to the occurrence of too frequent seizures, which obstructed extraction of a predefined duration (60 min) of data. Patient characteristics are shown in Table 1. The mean age of patients was 30.2 years (range, 13–46 years) and the mean epilepsy duration was 13.5 years (range, 3–43 years). Eight among the 10 patients were women. The etiology of epilepsy was hippocampal sclerosis in six patients (two were radiologically, whereas four were pathologically diagnosed), autoimmune encephalitis in one, unknown in two, and suspected low-grade glioma in one. We defined a temporal seizure as mesial onset if the earliest EEG change was observed in the deep three contacts of the depth electrodes, whereas as lateral onset if it was observed in the superficial three contacts. The SOZ was the mesial temporal lobe in seven patients, the lateral temporal lobe in one, and the mesial and lateral temporal lobe in two. We observed magnetic resonance imaging (MRI) abnormalities in nine patients. Hippocampal IED density ranged from 0.35 to 38.54/min and was above 5/min in 14 hemispheres. The right hemisphere of patient 8 was eliminated from analysis because of persistent artifacts in the P4 electrode. Ultimately, we analyzed the data from 13 hemispheres of 10 patients.

**Table 1:** Patient characteristics.

Patient	Age, years	Epilepsy duration, years	Sex	Etiology	Seizure onset zone	MRI findings	Lt IED density, per min	Rt IED density, per min	Resection (prognosis)	Pathology	ASMs at recording
1	46	43	M	HS	Lt mesial T, Rt lateral T	Lt Hipp atrophy, T2 hyperintensity	1.22	5.90	No		CBZ, LEV, VPA
2	31	5	F	Autoimmune encephalitis	Lt mesial T, Rt lateral T	Bil Hipp atrophy, T2 hyperintensity	9.22	9.36	No		CLB, VPA
3	32	12	F	Unknown	Rt mesial T	Bil Hipp atrophy	0.45	19.51	Yes (Engel III)	Gliosis	CLB, LTG
4	31	3	F	HS	Rt mesial T	Bil Hipp atrophy, T2 hyperintensity	1.52	8.41	Yes (Engel I)	HS	CBZ, LEV, TPM
5	42	12	F	HS	Rt mesial T	Bil Hipp atrophy, T2 hyperintensity	9.06	6.37	Yes (Engel I)	HS	None
6	24	14	M	Low-grade glioma (suspected)	Bil mesial T	Lt MTL enlargement, T2 hyperintensity	20.51	4.21	No		None
7	33	11	F	HS	Lt mesial T	Lt Hipp atrophy, Bil hipp T2 hyperintensity	11.97	11.10	No		None
8	13	9	F	HS	Lt mesial T	Lt Hipp atrophy, T2 hyperintensity	11.60	22.25	Yes (Engel I)	HS	None
9	26	6	F	Unknown	Lt lateral T	Rt Hipp atrophy	38.54	0.35	Yes (Engel I)	Gliosis	None
10	24	20	F	HS	Lt mesial T	Normal	21.52	0.94	Yes (Engel IV)	HS	None

Abbreviations: ASMs, anti-seizure medications; CBZ, carbamazepine; CLB, clobazam; F, female; Hipp, hippocampus; HS, hippocampal sclerosis; IED, interictal epileptiform discharge; LEV, levetiracetam; Lt, left; LTG, lamotrigine; M, male; MRI, magnetic resonance imaging; MTL, mesial temporal lobe; Rt, right; T, temporal; TPM, topiramate; VPA, valproate.

### 3.2 IED–spindle and IED–SO coupling analysis

**Fig. 2:**
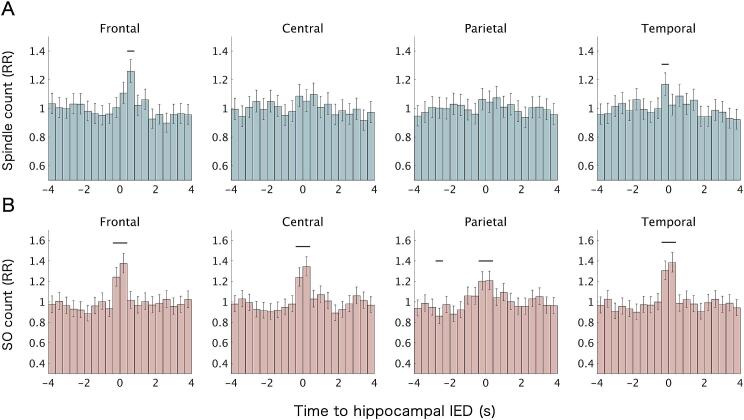
**Temporal relationship between hippocampal interictal epileptiform discharges (IEDs) and scalp spindles and slow oscillations (SOs).** (A) Peri-IED time histogram analysis of spindle onsets. (B) Peri-IED time histogram analysis of SO negative peaks. Both were evaluated using generalized linear mixed-effects models. Bars represent rate ratios (RRs) relative to the mean across all time bins, with error bars indicating 95% confidence intervals. Thick black horizontal lines indicate time bins with statistically significant increases in event occurrence compared to the overall mean (Bonferroni-corrected *p* < 0.05). Data are shown for ipsilateral frontal (F3/4), central (C3/4), parietal (P3/4), and temporal (F7/8) scalp electrodes.

The effect of time bins on spindle count was significant in the frontal (F3/4) (Wald *χ^2^* = 84.20, df = 19, Bonferroni-corrected *p* < 0.001) and temporal (F7/8) (*χ^2^* = 47.35, df = 19, Bonferroni-corrected *p* = 0.001) regions. *Post hoc* analyses revealed that spindle occurrence in the frontal region was significantly increased during the 0.4–0.8 s time bin following hippocampal IEDs, compared to the mean across all bins (rate ratio [RR] = 1.26, 95% confidence interval [CI] [1.18, 1.34], *Z* = 7.18, Bonferroni-corrected *p* < 0.001) (Fig. 2A). Similarly, spindle occurrence in the temporal region was elevated during the − 0.4–0 s time bin (RR = 1.17, 95% CI [1.09, 1.25], *Z* = 4.49, Bonferroni-corrected *p* < 0.001) (Fig. 2A).

Significant effects of time bins on SO count were observed in all four regions (frontal, *χ^2^* = 129.64, df = 19, Bonferroni-corrected *p* < 0.001; central, *χ^2^* = 133.19, df = 19, Bonferroni-corrected *p* < 0.001; parietal, *χ^2^* = 88.21, df = 19, Bonferroni-corrected *p* < 0.001; temporal, *χ^2^* = 158.15, df = 19, Bonferroni-corrected *p* < 0.001). *Post hoc* analyses revealed a significant increase in the incidence of SO negative peaks during the − 0.4–0 s (frontal, RR = 1.24, 95% CI [1.15, 1.34], *Z* = 5.78, Bonferroni-corrected *p* < 0.001; central, RR = 1.24, 95% CI [1.15, 1.33], *Z* = 5.82, Bonferroni-corrected *p* < 0.001; parietal, RR = 1.20, 95% CI [1.12, 1.29], *Z* = 4.88, Bonferroni-corrected *p* < 0.001; temporal, RR = 1.30, 95% CI [1.21, 1.40], *Z* = 7.35, Bonferroni-corrected *p* < 0.001) and 0–0.4 s (frontal, RR = 1.37, 95% CI [1.28, 1.47], *Z* = 8.92, Bonferroni-corrected *p* < 0.001; central, RR = 1.34, 95% CI [1.25, 1.44], *Z* = 8.30, Bonferroni-corrected *p* < 0.001; parietal, RR = 1.21, 95% CI [1.12, 1.30], *Z* = 5.00, Bonferroni-corrected *p* < 0.001; temporal, RR = 1.38, 95% CI [1.29, 1.48], *Z* = 9.23, Bonferroni-corrected *p* < 0.001) time bins, consistently across all four regions (Fig. 2B).

**Fig. 3:**
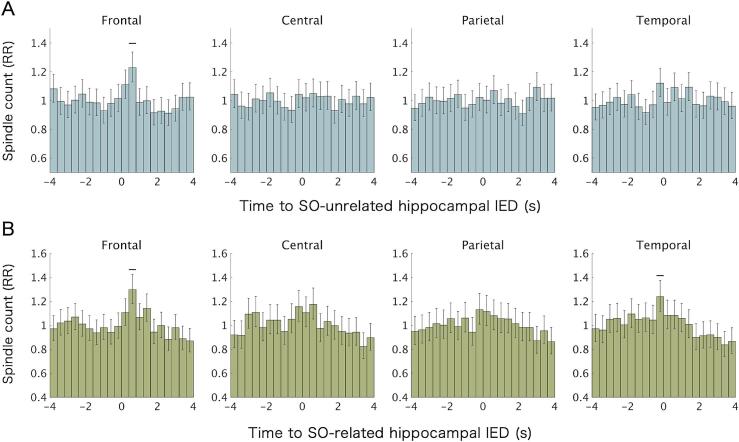
**Effects of slow oscillations (SOs) on the temporal relationship between hippocampal interictal epileptiform discharges (IEDs) and scalp spindles.** (A) Peri-IED spindle histogram analysis for SO-unrelated IEDs, defined as IEDs occurring more than 2.5 s away from any SO negative peak. (B) Peri-IED spindle histogram analysis for SO-related IEDs, defined as IEDs occurring within ± 2.5 s of an SO negative peak. Analyses were conducted using generalized linear mixed-effects models. Bars represent rate ratios (RRs) relative to the mean across all time bins, with error bars indicating 95% confidence intervals. Thick black horizontal lines indicate statistically significant deviations from the mean (Bonferroni-corrected *p* < 0.05). Data are shown for ipsilateral frontal (F3/4), central (C3/4), parietal (P3/4), and temporal (F7/8) scalp electrodes.

When SO-unrelated IEDs were analyzed separately, a significant effect of time bins was observed only in the frontal region (*χ^2^* = 43.89, df = 19, Bonferroni-corrected *p* = 0.004). *Post hoc* analysis showed a significant increase in spindle count in the 0.4–0.8 s time bin (RR = 1.23, 95% CI [1.13, 1.34], *Z* = 4.78, Bonferroni-corrected *p* < 0.001) (Fig. 3A). In contrast, when SO-related IEDs were analyzed separately, significant effects of time bins on spindle count were observed in the frontal (*χ^2^* = 60.67, df = 19, Bonferroni-corrected *p* < 0.001) and temporal (*χ^2^* = 48.89, df = 19, Bonferroni-corrected *p* = 0.001) regions. *Post hoc* analyses revealed increased spindle counts in the 0.4–0.8 s time bin in the frontal region (RR = 1.30, 95% CI [1.18, 1.42], *Z* = 5.47, Bonferroni-corrected *p* < 0.001) and in the − 0.4–0 s time bin in the temporal region (RR = 1.24, 95% CI [1.12, 1.37], Z = 4.04, Bonferroni-corrected *p* = 0.001) (Fig. 3B).

Prior experimental work in rodents has reported spindle-like activity associated with IEDs during wakefulness (Gelinas et al., 2016). We therefore visually inspected all artifact-free wake periods, but did not identify any clear frontal spindle-like activity.

### 3.3 Peri-IED spectral and amplitude analysis

**Fig. 4:**
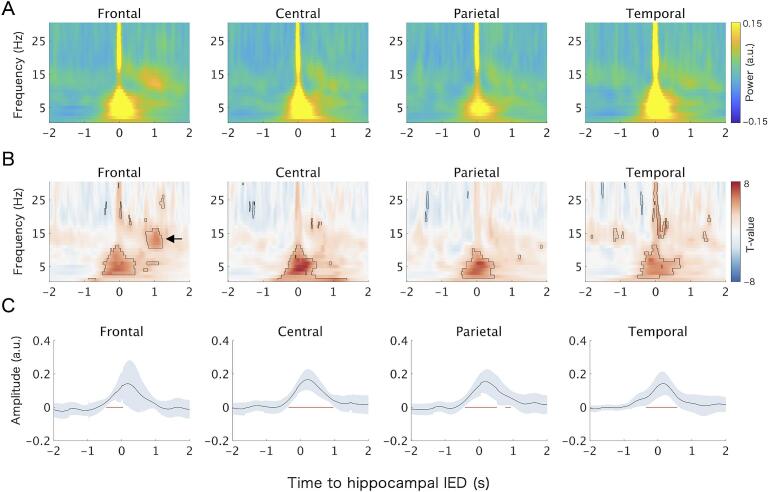
**Scalp electroencephalographic (EEG) changes associated with hippocampal interictal epileptiform discharges (IEDs).** (A, B) Results of *peri*-IED spectral analysis using linear mixed-effects models (LMMs). (A) Intercept (*β_0_*) maps represent the model-estimated average deviation in baseline-corrected, normalized power relative to the − 4 to − 2 s pre-IED interval. (B) Corresponding *t*-value maps from the statistical tests against zero. Black contours indicate time–frequency points with significant power changes (FDR-corrected *p* < 0.05). The arrow highlights a power increase in the spindle band, consistent with IED-induced spindles. (C) LMM-based analysis of *peri*-IED amplitude changes in the slow oscillation (SO) band (0.5–1.25 Hz). The black line shows the intercept (*β_0_*), representing the average deviation from baseline in z-normalized amplitude, with blue shading indicating 95% confidence interval. Thick red horizontal bars denote time points with statistically significant deviations from baseline (FDR-corrected *p* < 0.05). FDR, false discovery rate. (For interpretation of the references to colour in this figure legend, the reader is referred to the web version of this article.)

In the frontal region, we identified a time–frequency cluster of power increase between 0.75 and 1.23 s after the occurrence of hippocampal IEDs in the frequency range of 11–16 Hz (Fig. 4B, arrow), with the maximum *t* value observed at 1.08 s and 13 Hz (*β_0_* = 0.095, 95% CI [0.057, 0.134], *t*(11.26) = 5.47, FDR-corrected *p* = 0.009) (Fig. 4A, B). This frequency range was consistent with spindles (Fernandez and Lüthi, 2020). No similar findings were observed in the other regions (Fig. 4A, B). For the SO band (0.5–1.25 Hz), amplitude was significantly increased around the IED peak in all regions: frontal (−0.46 to 0.04 s), central (−0.35 to 0.97 s), parietal (−0.42 to 0.52 s), and temporal (−0.35 to 0.57 s) (Fig. 4C).

### 3.4 Impact of IED on spindle and spindle–SO coupling

No significant differences were observed in amplitude, duration, or frequency between IED-coupled and uncoupled spindles (Table 2). In addition, hippocampal IED density was not correlated with spindle density in any of the regions.

**Table 2:** Features of IED-coupled and −uncoupled spindles.

Variable	Region	Estimated marginal mean (95% CI)		Statistical results	
		IED-coupled spindles	IED-uncoupled spindles	*β* (95% CI)	Bonferroni-corrected *p* value
Frequency (Hz)	Frontal	12.535 (12.078–12.992)	12.522 (12.066–12.979)	0.012 (−0.040–0.064)	1.000
	Central	12.586 (12.214–12.959)	12.610 (12.240–12.981)	−0.024 (−0.097–0.049)	1.000
	Parietal	12.891 (12.337–13.445)	12.870 (12.317–13.422)	0.021 (−0.054–0.097)	1.000
	Temporal	12.954 (12.435–13.473)	12.892 (12.374–13.409)	0.062 (−0.006–0.131)	0.301
Normalized amplitude (a.u.)	Frontal	3.035 (2.916–3.155)	2.955 (2.842–3.069)	0.080 (0.014–0.146)	0.072
	Central	3.111 (3.006–3.216)	3.032 (2.936–3.128)	0.079 (0.008–0.149)	0.115
	Parietal	3.083 (2.946–3.219)	3.045 (2.914–3.176)	0.037 (−0.030–0.105)	1.000
	Temporal	3.026 (2.886–3.165)	2.953 (2.818–3.087)	0.073 (0.006–0.140)	0.129
Duration (s)	Frontal	0.934 (0.880–0.988)	0.922 (0.868–0.975)	0.012 (−0.007–0.031)	0.827
	Central	0.847 (0.814–0.880)	0.847 (0.816–0.878)	0.000 (−0.019–0.019)	1.000
	Parietal	0.864 (0.827–0.901)	0.865 (0.829–0.901)	−0.001 (−0.020–0.018)	1.000
	Temporal	0.863 (0.827–0.899)	0.869 (0.835–0.903)	−0.006 (−0.025–0.014)	1.000

Abbreviations: IED, interictal epileptiform discharge; a.u., arbitrary unit.

**Fig. 5:**
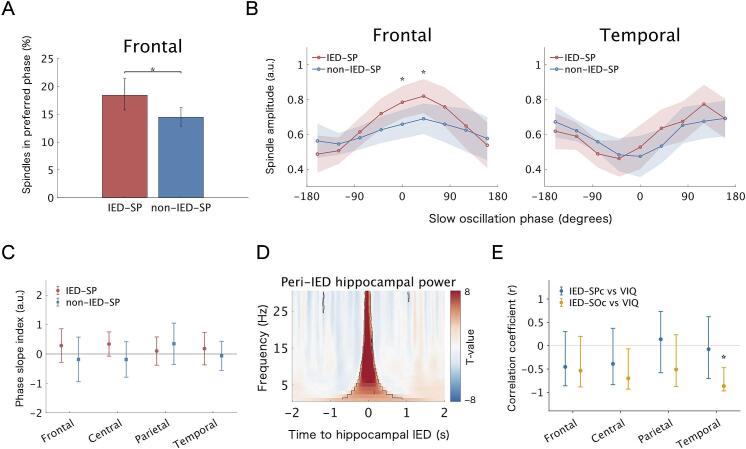
**Effects of hippocampal interictal epileptiform discharges (IEDs) on spindle–slow oscillation (SO) coupling, *peri*-IED spectral changes, and neuropsychological correlations.** (A) Ratio of spindles occurring within the preferred SO phase in the frontal region, estimated using a generalized linear mixed-effects model. This metric reflects the phase consistency of spindle timing relative to SOs. Error bars represent 95% confidence intervals (CIs). The asterisk indicates a significant difference between IED-coupled and uncoupled spindles (Bonferroni-corrected *p* < 0.05). (B) Spindle amplitude modulation by SO phase in the frontal and temporal regions, estimated by linear mixed-effects models (LMMs). Solid lines show the marginal means of z-scored spindle amplitude for each SO phase bin; shaded areas denote the 95% CIs. Asterisks indicate significant differences between IED-coupled and uncoupled spindles within specific phase bins (Bonferroni-corrected *p* < 0.05). (C) Directionality analysis between SO phase and spindle amplitude using the phase-slope index (PSI). Positive values indicate directionality from SO phase to spindle amplitude. Error bars indicate the 95% CIs. (D) Peri-IED spectral changes in hippocampal electrodes. The t-value map was obtained from *peri*-IED spectral analysis using an LMM. Black contours indicate time–frequency points with significant power changes relative to baseline (−4 to − 2 s) (FDR-corrected *p* < 0.05). (E) Correlations of verbal intelligence quotient (VIQ) with IED–spindle and IED–SO coupling strength. Error bars indicate the 95% CIs. Asterisks indicate significant associations (Bonferroni-corrected *p* < 0.05). IED-SP, IED-coupled spindle; IED–SOc, IED–SO coupling strength; IED–SPc, IED–spindle coupling strength; non-IED-SP, IED-uncoupled spindle.

The ratio of spindles within the preferred SO phase was greater in IED-coupled than in uncoupled spindles in the frontal region (odds ratio = 1.34, 95% CI [1.08, 1.65], *Z* = 2.67, Bonferroni-corrected *p* = 0.030) (Fig. 5A). This effect was not observed in any other regions. Regarding spindle amplitude modulation, the interaction between SO phase and spindle type (IED-coupled vs. uncoupled) was significant in the frontal (*F*(8, 26113.6) = 3.98, Bonferroni-corrected *p* < 0.001) and temporal (*F*(8, 22450.6) = 2.74, Bonferroni-corrected *p* = 0.020) regions. *Post hoc* analyses revealed that in the frontal region, IED-coupled spindles showed greater amplitude than that of uncoupled spindles within the − 20° to 20° (*t*(2937.2) = 3.33, Bonferroni-corrected *p* = 0.008) and 20° to 60° (*t*(2966.4) = 3.30, Bonferroni-corrected *p* = 0.009) phase bins, where the amplitude of both types was higher than in other bins (Fig. 5B). In contrast, no significant *post hoc* difference was found between spindle types in the temporal region (Fig. 5B).

In the CFD analysis, PSI values did not significantly differ from zero in any region for either IED-coupled or IED-uncoupled spindles (all Bonferroni-corrected *p* > 0.05) (Fig. 5C).

### 3.5 Impact of SOZ location on spindle and SO density

In the exploratory analysis of SOZ location, we did not observe a clear difference in scalp spindle density or SO density between hemispheres with mesial versus lateral temporal SOZ across ipsilateral scalp regions (mesial: n = 10 hemispheres; lateral: n = 3 hemispheres; all Bonferroni-corrected *p* > 0.05).

### 3.6 Peri-IED hippocampal EEG analysis

Peri-IED spectral analysis of hippocampal EEG showed no significant increase in spindle-band power within ± 2 s of hippocampal IEDs (all FDR-corrected *p* > 0.05) (Fig. 5D).

### 3.7 Correlations between neuropsychological outcomes and IED–spindle or IED–SO coupling

FIQ, VIQ, and PIQ were available for 9 of the 10 patients and were obtained using the WAIS-R (1 patient), WAIS-III (7 patients), or WISC-III (1 patient). Among the exploratory correlation analyses, temporal IED–SO coupling strength showed a significant negative correlation with VIQ (*r* =  − 0.86, 95% CI [−0.97, −0.47], Bonferroni-corrected *p* = 0.010) (Fig. 5E), but not with FIQ or PIQ. No significant correlations were observed between IED–SO coupling strength in the other regions or IED–spindle coupling strength in any region and FIQ, VIQ, or PIQ.

## 4 Discussion

To the best of our knowledge, this study represents the first systematic investigation of the association between directly recorded hippocampal IEDs and cortical spindles and SOs in a homogeneous group of patients with TLE.

Our findings indicate that hippocampal IEDs induce cortical spindles, with this effect being most prominent in the frontal region. In this region, spindle occurrence increased following IEDs regardless of their temporal relationship to SOs, suggesting a direct, IED-driven mechanism of spindle generation. Supporting this, *peri*-IED spectral analyses revealed a significant increase in spindle-band power in the frontal region after IEDs. In contrast, spindles increased prior to IEDs in the temporal region, but only for SO-related events, and without a corresponding increase in spindle-band power. This pattern suggests that the apparent coupling in the temporal region may reflect concurrent SO activity rather than a direct IED effect, or possibly the misclassification of IEDs as spindles by the detection algorithm. Regarding IED–SO coupling, IEDs occurred more frequently around the negative peaks of SOs across all cortical regions, consistent with previous reports. This finding was further supported by complementary *peri*-IED amplitude analyses. Finally, two independent analyses consistently showed that spindle–SO coupling was stronger for IED-coupled than for uncoupled spindles, particularly in the frontal region and to a lesser extent in the temporal region. Although frontal spindle occurrence increased after hippocampal IEDs, IED-coupled and IED-uncoupled spindles did not significantly differ in frequency, duration, or overall amplitude. Thus, in our dataset, hippocampal IEDs seemed to affect mainly the timing of frontal spindles and their coupling with SOs, rather than the basic features of the spindles themselves.

To our knowledge, this is the first study to demonstrate that hippocampal IEDs induce cortical spindles at the group level in a homogeneous population of patients with TLE. Previous reports have described similar findings, but often with small or heterogeneous samples. For example, Gelinas et al. (2016) observed frontal spindles induced by IEDs in four patients, but only one had hippocampal IEDs (Gelinas et al., 2016). Dahal et al. (2019) found that IEDs from various limbic regions induced cortical spindles in 10 patients with focal epilepsy (Dahal et al., 2019). Our findings differ from those of Sákovics et al. (2022), who examined 21 patients with focal epilepsy (including 14 with TLE) using foramen ovale electrodes (Sákovics et al., 2022). They reported IED–spindle coupling in 52% of patients, but the direction of coupling (i.e., whether IEDs preceded or followed spindles) was inconsistent. Several methodological differences may explain this discrepancy. First, we used depth electrodes for direct hippocampal recordings, while foramen ovale electrodes may capture signals from multiple regions. Second, we focused solely on patients with TLE, reducing variability in epileptogenic zones. Third, we analyzed hemispheres separately, whereas Sákovics et al. combined ipsilateral and contralateral data, possibly obscuring lateralized effects.

The mechanisms underlying IED-induced spindle generation remain unclear. Gelinas et al. (2016) observed reduced cortical firing prior to IED-induced spindles, a pattern similar to that seen following electrical or magnetic cortical stimulation, which induces slow waves followed by spindles (Massimini et al., 2007, Vyazovskiy et al., 2009). These findings suggest one possible mechanism: a strong electrical input may transiently suppress cortical neuronal activity, which could promote conditions favorable for subsequent spindle generation as part of an intrinsic cortical response. This hypothesis aligns with our results and may explain the frontal predominance of IED-induced spindles, given that hippocampal stimulation inhibits firing in the prefrontal cortex (Girardeau et al., 2009), potentially through monosynaptic projections from the hippocampus to the prefrontal cortex (Godsil et al., 2013). An alternative hypothesis posits that IED–spindle coupling is not causal but reflects a shared temporal relationship with SOs (von Ellenrieder et al., 2020). Indeed, prior studies have shown that IEDs tend to occur around the negative peak of SOs (Chen et al., 2023, Frauscher et al., 2015b, Ujma et al., 2017), while spindles preferentially align with the positive peak (Clemens et al., 2007). A recent study by Wodeyar et al. (2024) further demonstrated that cortical IEDs and thalamic spindles are phase-locked to distinct phases of SOs (Wodeyar et al., 2024). However, our findings are not fully accounted for by this hypothesis. While IED–spindle coupling in the temporal region appeared to be SO-dependent, the coupling in the frontal region remained significant even after excluding SO-related IEDs. These findings suggest that both mechanisms—direct induction of spindles by IEDs and indirect coupling mediated by shared alignment with SOs—may coexist. The relative contribution of each mechanism likely depends on factors such as the anatomical origin of IEDs, the region in which spindles are recorded, and individual patient characteristics.

Spindle–SO coupling plays a critical role in physiological processes such as memory consolidation (Helfrich et al., 2021). We initially hypothesized that spindles induced by IEDs might emerge at non-physiological timings, potentially weakening their synchronization with SOs. However, our findings revealed the opposite. IED-coupled spindles occurred more frequently near the preferred SO phases and showed greater amplitude modulation by SOs than did uncoupled spindles, particularly in the frontal region. These results suggest that spindle–SO coupling is actually stronger for IED-coupled spindles. This implies that IEDs may possess the capacity to entrain ongoing oscillatory dynamics during sleep. Although the precise mechanism remains uncertain, one plausible explanation is that IEDs transiently suppress cortical firing (Gelinas et al., 2016), thereby resetting the phase of ongoing SOs and enhancing their temporal alignment with the subsequent IED-induced spindles. In the CFD analysis, PSI values did not significantly differ from zero, suggesting no consistent directionality of spindle–SO coupling across patients. In healthy young adults, non-zero PSI values consistent with an SO-to-spindle direction have been reported across widespread regions, whereas this pattern was less apparent in some regions in older adults (Helfrich et al., 2018). Our findings may thus be compatible with disease-related disruption of spindle–SO directionality, similar to age-dependent changes.

Our findings may have important implications for understanding memory consolidation impairment in patients with TLE. Notably, we observed no correlation between the densities of IEDs and spindles, suggesting that IED-induced spindles may not simply add to, but rather replace physiological spindles. This potential substitution raises concerns about disrupted sleep-dependent memory processing. The frontal predominance of IED-induced spindles is particularly noteworthy in light of the known role of hippocampal–prefrontal interactions in memory consolidation. In both rodents and humans, hippocampal ripples coordinate with frontal spindles and SOs, facilitating memory transfer during sleep (Gelinas et al., 2016, Helfrich et al., 2019, Maingret et al., 2016, Peyrache et al., 2011). Our finding that hippocampal IEDs preferentially induce spindles in the frontal cortex suggests that these pathological events may hijack this physiological communication. Moreover, we found that spindle–SO coupling was stronger in IED-coupled than in uncoupled spindles, particularly in the frontal region. While such coupling is typically beneficial for memory consolidation, its enhancement by IEDs may reflect maladaptive plasticity, wherein epileptiform discharges co-opt normal sleep rhythms for pathological synchronization. Consistent with this possibility, our exploratory analysis further showed that stronger temporal IED–SO coupling was associated with lower VIQ. This observation is preliminary, given the small sample size and the heterogeneity of the available neuropsychological data, but it suggests that IED-related disruption of physiological sleep oscillatory dynamics may be clinically relevant. Because standardized memory measures were not uniformly available, future studies will be needed to determine whether abnormal IED–spindle and IED–SO coupling are related to memory dysfunction in patients with TLE.

In addition to these mechanistic and functional insights, we have demonstrated that hippocampal IEDs have specific remote effects on scalp-recorded spindles. These findings may improve the recent attempt at noninvasive detection of hippocampal epileptic discharges, often invisible on scalp EEG, using artificial intelligence (AI) (Abou Jaoude et al., 2022, Lam et al., 2016).

## 5 Limitations

First, as with previous studies investigating IED-induced spindles using intracranial EEG (Dahal et al., 2019, Gelinas et al., 2016, Wodeyar et al., 2024), the sample size was relatively small. Therefore, the generalizability of our findings remains limited and should be validated in future studies with larger cohorts. In particular, the IED-coupled versus IED-uncoupled analyses involve a two-condition comparison, which generally requires more information (i.e., a larger sample) to estimate group differences with high precision than analyses assessing a single condition; accordingly, these results should be interpreted with appropriate caution. Second, owing to the study’s retrospective design, we were unable to assess memory consolidation performance or examine its relationship with the observed electrophysiological changes. As a result, this study does not directly address whether IED-induced spindles contribute to cognitive impairment in patients with TLE. Third, intracranial sampling was primarily limited to hippocampal contacts, and adjacent mesial temporal lobe structures, including the amygdala, were not systematically sampled. Therefore, “hippocampal IEDs” should be interpreted as IEDs recorded in hippocampal contacts rather than precise source localization of the IEDs. Accordingly, we cannot exclude the possibility that some IEDs recorded in hippocampal contacts reflected propagated activity from nearby mesial temporal lobe structures. Fourth, automatic spindle detection is known to be sensitive to the choice of algorithm (Warby et al., 2014). Our detection method was developed and validated in healthy individuals and may either underestimate or overestimate spindle events in the context of interictal epileptiform activity (Kramer et al., 2021). Nevertheless, we believe that this had a limited impact on our primary findings, as our complementary spectral analyses showed a consistent increase in spindle-band power following IEDs. Finally, anti-seizure medication status during monitoring was heterogeneous and not experimentally controlled (Table 1). While most patients were off ASMs (6/10), a subset remained on ASMs (4/10), including clobazam in two patients, and these medications could have modulated sleep spindle characteristics and spindle–SO coupling, potentially contributing to inter-individual variability.

## 6 Conclusions

This study demonstrated that hippocampal IEDs induce cortical spindles, most prominently in the frontal region, likely through a direct mechanism. In contrast, coupling observed in the temporal region may be mediated by concurrent SOs. Notably, IED-coupled spindles exhibited stronger synchronization with SOs than did uncoupled spindles, particularly in the frontal regions, suggesting that IEDs can entrain ongoing sleep-related oscillations. These IED-induced spindles may replace rather than supplement physiological ones, potentially disrupting hippocampal–cortical communication. Our findings highlight a novel mechanism by which interictal activity could impair cognitive function in patients with TLE. Moreover, the observed scalp EEG signatures of hippocampal IEDs may inform future noninvasive approaches for detecting deep epileptic discharges, including AI-assisted methods. Further studies with larger cohorts and behavioral assessments are warranted to validate and extend these results.

## Author contributions

T.U., S.T., N.I., and J.K. conceived and designed the study. H.S., T.M., T.O., N.M., and S.A. contributed to data acquisition. T.U., E.A.B., and S.A. analyzed the data. T.U., H.M., and J.K. drafted the manuscript.

## Funding

This work was supported in part by a research grant from Japan Epilepsy Research Foundation (No. JERF TENKAN 24002) and by a Grant-in-Aid for Scientists (Nos. 19K07964 and 24K10648) from the Ministry of Education, Culture, Sports, Science, and Technology in Japan.

## Declaration of competing interest

The authors declare that they have no known competing financial interests or personal relationships that could have appeared to influence the work reported in this paper.
